# Cardiac magnetic resonance imaging for comprehensive assessment of bicuspid aortic valve: comparison with transthoracic echocardiography, dual-source CT and operative findings

**DOI:** 10.1186/1532-429X-13-S1-P339

**Published:** 2011-02-02

**Authors:** SungMin Ko

**Affiliations:** 1 Konkuk University Hospital, Seoul, Korea, Republic of

## Introduction

Patients with bicuspid aortic valve (BAV) are at increased risk for both valvular and vascular complications. Preoperative knowledge about AV morphology, the presence and extent of AV and annulus calcification, valvular complications, and ascending aorta (AA) diameter is very important for surgical planning in patients with BAV.

## Purpose

To evaluate the value of cardiac magnetic resonance imaging (CMR) for the comprehensive assessment BAV in comparison with transthoracic echocardiography (TTE), dual-source computed tomography (DSCT), and operative findings.

## Methods

Seventy-three consecutive patients (49 men, mean age 51.2 years) with BAV who underwent TTE, DSCT, cardiac MRI and valve surgery were enrolled in this study. All CMR studies were performed with a 1.5 T whole-body system using an 8-element phased array surface coil. Two independent radiologists assessed the type of BAV (classified according to number of raphe), functional status of the BAV, AA diameter and left ventricular ejection fraction (LVEF). Stenotic aortic valve area (AVA) was computed with the continuity equation on TTE and by direct planimetry on DSCT and CMR. We compared quantitative grading of aortic regurgitation (AR) by CMRI with semiquantitative grading of AR by TTE. AA diameter was measured at 4 levels from mid-systole on DSCT and CMR. LVEF by CMR (Simpson’s method) was compared with the use of TTE (M-mode method).

## Results

Patients underwent AV repair (n= 72) or replacement (n=1) with AA graft replacement (n=3) or wrapping (n=28). CMR showed 91.8% agreement of BAV type compared with operative findings [no raphe (n=30) and raphe (n=43)]. CMR showed excellent agreement (*κ*=0.954) of the underlying BAV complications as compared with TTE [regurgitation (n=21), stenosis (n=27), and mixed (n=24)]. Stenotic AVA (n=51) by CMR (0.88±0.29 cm^2^) correlated well with AVA by DSCT (1.04±0.30 cm^2^, *r* =0.84, *p*<0.0001) and TTE (0.82±0.25 cm^2^, *r*=0.75, *p* <0.0001). Regurgitation grading by CMR was significantly correlated with the grading of AR severity by TTE (n=45, *r*=0.85, *p* <0.0001). AA dilatation more than 4.5 cm in diameter was present in 30 (41%) patients. There was excellent correlation (*r*=0.98, *p*<0.0001) in the mean diameter of AA between DSCT and CMR (35.0±8.1 mm vs 35.2±8.0 mm, *p*=0.061). A moderate correlation between CMR and TTE was shown for the evaluation of LVEF (56.2±10.2% vs 62.3±12.4%, respectively; *r*=0.63).

## Conclusions

**C**MR allows accurate imaging technique for comprehensive assessment of patients with BAV.

